# Additive yield response of chickpea (*Cicer arietinum* L.) to rhizobium inoculation and phosphorus fertilizer across smallholder farms in Ethiopia

**DOI:** 10.1016/j.agee.2018.01.035

**Published:** 2018-07-01

**Authors:** Endalkachew Wolde-meskel, Joost van Heerwaarden, Birhan Abdulkadir, Sofia Kassa, Ibsa Aliyi, Tulu Degefu, Kissi Wakweya, Fred Kanampiu, Ken E. Giller

**Affiliations:** a International Livestock Research Institute, P.O. Box 5689, Addis Ababa, Ethiopia; b Plant Production Systems, Wageningen University, P.O. Box 430, 6700 AK, Wageningen, The Netherlands; c Ethiopian Institute of Agricultural Research, Debre-Zeit Agricultural Research Center, Bishoftu, Ethiopia; d School of Plant Sciences, Haramaya University, Haramaya, Ethiopia; e International Crop Research Institute for SemiArid Tropics, Addis Ababa, Ethiopia; f Oromia Agricultural Research Institute, Robe, Ethiopia; g International Institute of Tropical Agriculture, Nairobi, Kenya

**Keywords:** Nitrogen fixation, Grain legume, Mesorhizobium, Yield gaps, Yield variability

## Abstract

The impacts of rhizobium inoculation on growth and yield of chickpea have mainly been tested in experiments conducted in greenhouses or on research stations. We report the response of the crop to inoculation (I) and phosphorus fertilizer (P) application across a large number of smallholder’s farms over four regions of Ethiopia, covering diverse soil fertility and agro-ecological conditions. Increased grain yields due to the soil fertility treatments was evident for 99% target farmers. On average, I and P increased grain yield by 21% and 25% respectively, while the combined application of I and P resulted in a 38% increase. However, observed grain yields on control plots and responses to the treatments on individual farms varied greatly, and relative yield responses (%; yield of P and/I minus control yield, divided by control yield) ranged from 3% to 138%. With the exception of a few extremely poorly yielding locations, average responses to P and I were high across a wide range of control yields, indicating the possibility of boosting chickpea productivity for smallholders with P fertilizer and inoculant technology. Variation in response to rhizobium inoculation was mostly independent of agro-ecology and soil type although it was found to be low on a number of farms with extremely high N contents (%). Assuming that a relative yield increase of 10% due to treatment effects is required to be visible, 71%, 73% and 92% of the farmers observed a yield benefit by applying P, I, and P + I, respectively. The results are discussed with respect to the additive benefits of P fertilizers and rhizobial inoculation and their implications for wide scale promotion of inoculant technology to smallholders.

## Highlights

• On MPN assessment, native rhizobium population size on the study site was less than 10 cfu g^−1^ of soil.
• On a wide range of on-farm trials, inoculation (I) increased chickpea grain yields for 99% of target farmers.
• I and P fertilizer in combination, resulted on average, in a 38% yield increase over the control plots.
• Variation in response to rhizobium inoculation was mostly independent of agro-ecology and soil type.
• Rhizobial inoculation offers a cheap and highly effective means for the sustainable intensification of smallholder agriculture.

## 1 Introduction

Chickpea (*Cicer arietinum* L.) is globally the third most important food legume after common bean and soybean (Namvar and Sharifi, 2011). It is widely cultivated by smallholders in Mediterranean and semi-arid climates but in Africa is largely restricted to the cool highlands of Ethiopia (Anbessa and Bejiga, 2002). However, it also grows in Sudan under irrigation and rain-fed systems and is an increasingly important crop in Tanzania. In 2014, Ethiopia produced almost 60% of Africa’s total chickpea (FAOSTAT, 2014; Ojiewo, 2016). The total area of chickpea in Ethiopia has increased from 168,000–230,000 ha over the past decade (CSA, 2014), with desi varieties grown mainly for the local market and the larger seeded, Kabuli varieties largely for export. Yet productivity of chickpea remains low, with national average yield of 1.7 t ha^−1^ (BTL, 2013; FAOSTAT, 2014; CSA, 2016, CSA, 2017), far below the potential yield of 4–5 t ha^−1^ reported on experimental stations (Bejiga and van der Maesen, 2006; Fikre, 2016).

Chickpea occupies an important position amongst the pulse crops grown in Ethiopia because of its multiple functions. It is a key component of the daily diet, and thus an important protein source for Ethiopian households who cannot afford animal products. Chickpea residue, locally known as “Defeka”, is important as a feed resource for livestock during the dry months of the year when green fodder is unavailable. This helps farmers to keep robust oxen whose draught power is critical for land preparation at the onset of the rains. Chickpea plays an important role in Ethiopia’s foreign exchange earnings through export to Asia and Europe (Shiferaw and Teklewold, 2007). Another attractive feature of chickpea is its ability to fix atmospheric nitrogen in symbiosis with rhizobia, contributing directly to grain protein and reducing the need for N fertilizer for subsequent crops. It thereby has great potential to improve soil N status (Tena et al., 2016; Ben Romdhane et al., 2008; Funga et al., 2016; Khaitov et al., 2016) and is an ideal candidate for intensification of the tef monoculture that is common in Ethiopia. Chickpea is produced mainly in the central, northern and north western highland areas at elevations of 1400–2400 m above sea level where annual rainfall ranges from 700 and 2000 mm. It is often grown after the cereals wheat (*Triticum* spp.) and tef (*Eragrostis tef* (Zucc.) Trotter) are harvested on vertisols using residual moisture which extends the cropping season through September–December. As a result, growing chickpea allows the farmers to produce extra crop on the same land.

Legume yields and nitrogen fixation depends on the genotype of the legume (G_L_), the rhizobium strain (G_R_) and the interactions of these with the bio-physical environment (E), and management practices (M) expressed as the interaction: (G_L_ × G_R_) × E × M (Giller et al., 2013). Research efforts have focused on breeding (MoARD, 2009; Jarso et al., 2011; BTL, 2013). The use of agrochemical inputs with legumes remains limited in Africa (Chianu et al., 2011), and chickpea is grown without fertilizer often on marginal lands in Ethiopia, with a common notion among farmers that legume crops do not need nutrient inputs. Yet poor legume yields are often a reflection of poor soil fertility (Franke et al., 2016).

Chickpea can fix 60–80% of its nitrogen requirement (SPG, 2016; Giller, 2001), amounting to 60–176 kg N ha^−1^ (Beck et al., 1991; Shiferaw et al., 2004). It is selective in its symbiotic requirement, nodulating with only a specific group of rhizobium species (Tena et al., 2016; SPG, 2016). The absence of compatible strains and the small rhizobial population in the soil are important limitations for nodule formation in chickpea (Kantar et al., 2010). Inoculation with effective strains at planting time is recommended if the soil population density of compatible rhizobia is less than 50 cells per gram of soil (Thies et al., 1991a, Thies et al., 1991b). There is increasing evidence to suggest that inoculation enhances plant growth, grain and biomass yield in chickpea (Ben Romdhane et al., 2008; Funga et al., 2016; Khaitov et al., 2016; Tena et al., 2016). In Ethiopia, experiments to date examining the effect of rhizobium inoculation on chickpea growth and yields have been largely restricted to greenhouses and research stations. Here, we report the responses of chickpea to inoculation (I) and phosphorus fertilizer (P) application from widespread testing on smallholder farmers’ fields. Our central aim was to understand the effect of the treatments (I, P and/or I + P) on grain yields of the crop across a large number of smallholders’ plots representing diverse soil fertility and agro-ecological conditions. To this end we conducted simple trials on more than 100 farmers’ fields in four Woredas (districts) in central, south and south-east Ethiopia. In addition, the variation in response to the soil fertility treatments across individual farms were explored to identify whether we could identify variables that explain the occurrence and magnitude of response. Such knowledge is important to assist both in targeting of the technologies and to identify the need for further research on rehabilitation measures.

## 2 Materials and methods

### 2.1 The test environment

**Fig. 1:**
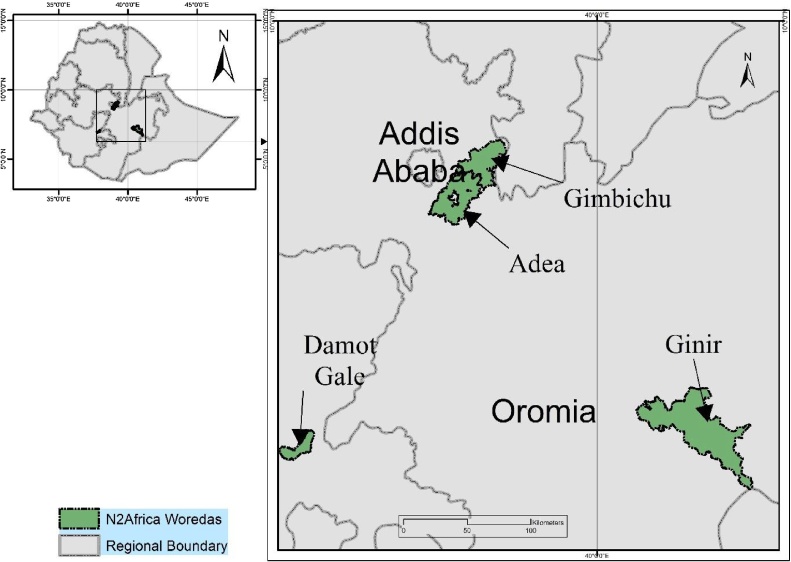
Map showing the Woredas where the on-farm trials were conducted (2012–2015).

On-farm demonstration trials were conducted over four cropping seasons from 2012 to 2015, in four different Woredas (districts), namely Ada’a/Gimbichu (Central), Damote-Gale (South), and Ginir (South-east Ethiopia) (Fig. 1). The sites are located within an elevational range of 1860–2493 m above sea level, with annual mean temperature range of 18–19.5 °C, and annual mean rainfall of 815–1255 mm (Table 1). There was a significant drought in 2015 which was an El Niño year. The climates at Ada’a/Gimbichu and Giner are characterized as “hot to warm sub humid” while Damote-Gale is “Hot to warm moist”. T’eff (*Eragrostis tef* (Zucc.) Trotter), wheat (*Triticum* spp.) and maize (*Zea mays* L.) are the most important cereal crops, but the crop mix varies among locations with more diversity at Damote-Gale (Table 1). All Woredas have a mixed farming system with crops and livestock. In all cases, chickpea is grown on residual moisture immediately following the main crop harvest – wheat (Ginir), teff (Ada’a/Gimbichu) and maize and/or tubers and spices (Damote-Gale). The soils of the trial sites are Eutric Vertisols at Ada’a/Gimbichu and Giner while Humic Nitisols are dominant at Damote-Gale (Table 1).

**Table 1:** Agro-ecological characteristics of the study Woredas.

	Central (Ada'a/Gimbichu)	Southern (Damot Gale)	South-eastern (Ginir)
Agroecological zone^a^	Hot to warm sub humid	Hot to warm moist	Hot to warm sub humid
Dominant soil type^b^	Eutric Vertisols	Humic Nitisols	Eutric Vertisols
Annual mean rainfall (mm)^c^	815	1127	1254
Annual mean min T (°C)^c^	10.5	13.8	13.3
Annual mean max T (°C)^c^	25.5	25.1	24.6
Annual mean T (°C)^c^	18.0	19.5	19.0
Rainfall (mm), in the year of experimentation			
2012	726	1033	1174
2013	738	1487	1450
2014	762	1215	1426
2015	687	776	967
Main crops	Teff, wheat, chickpea	Maize, sweet potato, common bean	Wheat, barley, teff, black cumin

±Weather data observed (Eth. Meteorological services).

a MoARD (2009).

b Harmonized Soil Database, FAO (2012).

c NMA: long-term mean rainfall and temperature.

### 2.2 On-farm demonstration trials

In 2012 and 2013, 23 farmers participated in on-farm demonstration trials. In the 2014 and 2015 seasons, the number of farmers participating increased to 93. The demonstration trials served for learning about improved chickpea technology packages through farmers’ field days, visits and technology evaluation events organized for large number of farmers from adjoining Kebeles (villages). Plots were selected to be accessible for farmers and visible to passers-by. The trials were not replicated on each farm, but farms were considered as replicates (Table 2).

**Table 2:** Chickpea varieties used in the on-farm trials from 2012 to 2015 in Ethiopia.

Variety/cultivar name	Type	Adaptation Elevation Range (masl)	Maturity days	Seed color	Grain yield (t ha^−1^)	
					Potential^a^	On-farm^a^
Arerti	Kabuli	1900–2600	105–155	White	2.6–4.6	2.0–3.2
Habru	Kabuli	1800–2600	91–150	White	2.4–3.2	–
Natoli	Desi	1800–2700	88–142	Light golden	1.1–4.6	3.5–3.7

a 0–100 kg ha^−1^ DAP (NPK: 18:46:0), no inoculation.

Trials included four treatments; an uninoculated and unfertilized control plot (C), a plot inoculated with rhizobium (I), inoculated but with phosphorus fertilizer (P), and both rhizobium inoculated and phosphorus fertilized (P + I). Due to lack of availability, the phosphorus fertilizers sources differ over the seasons, and were TSP (tri-super phosphate) in 2012, DAP (di-ammonium phosphate) in 2013–2014 and an NPS blend in 2015. The rate was held constant at 23 P_2_O_5_ kg ha^−1^ (13 kg P ha^−1^) applied at sowing. Altogether, three improved chickpea varieties (two Kabuli type – Arerti and Habru and one Desi type – Natoli) were selected on the basis of local adaptation and market preferences (Table 2). Natoli and Habru are short to medium maturity (88–150 days) while the Arerti has relatively long duration (105–155 days). Seed was sown in rows 30–40 cm apart, spaced 10 cm within rows. Each treatment plot measured 10 m by 10 m with 1 m paths separating the plots.

Inoculants were purchased from the sole commercial inoculant producer in Ethiopia: Menagesha Biotech Industry PLC, Addis Ababa. The inoculants used lignite as a carrier and two chickpea *Mesorhizobium* strains (CP-41 in 2012 and CP-029 in 2013–2015). These strains have been tested under a wide range of ecological conditions in Ethiopia (Tena et al., 2016; Funga et al., 2016). Seeds were inoculated at a rate of 5 g of inoculant per kg of seed using sugar solution as a sticker. Inoculation was done under shade and the inoculated seed was kept for few minutes until air dry before planting. In all farms, uninoculated treatments plots were sown first to avoid cross contamination.

Composite soil samples were collected before planting at depth of 0–20 cm sampled at 13 even intervals in a “W” pattern throughout the field. The samples were weighed, air-dried, ground to pass through a 2 mm sieve before analysis. Soils were analysed following standard laboratory procedures in soil laboratories in Ethiopia (2012–2014) and at IITA at Ibadan, Nigeria (2015) for pH (1:1 soil to H_2_O), organic C (Walkley–Black), total N (Kjeldahl), P Mehlich, and exchangeable K, Ca and Mg according to standard procedures (IITA, 1982). The composite soil sample from each field at Damote-Gale was used to determine the population of rhizobia compatible to chickpea following most-probable-number (MPN) plant infection count (Somasegaran and Hoben, 1994) during the 2012 crop season.

Plots were sown between mid-August and late-September and managed by the farmers assisted by a development agent (DA) or field technician, who were responsible for keeping records of different operations. Nodulation was assessed at mid flowering growth stage at Damote (in 2012 and 2014) from randomly sampled ten plants in the plot at each of the respective treatments. The plants were uprooted carefully, washed and the number of nodules recorded. At the end of the season, each plot was harvested discarding single border rows at each edge. The total biomass (the grain and stover) was weighed at harvest and the grain (after threshing) weighed separately. Plants were sampled randomly from 28 farms in each season. At physiological maturity, five non-border plants were sampled from the respective treatment plots, separated into grain and straw, oven dried at 70 °C to a constant weight and ground to pass through a 1 mm sieve prior to analysis for N (semi-micro Kjeldahl digestion followed by distillation).

### 2.3 Statistical analysis

It was not possible to measure all variables at all locations and/or growing seasons. A total of 144 trials were established, but reliable yield data was recorded for only 107 trials. As geographic location of trials was largely confounded with each year, a year/location factor was created by assigning a unique level to each specific combination of year and location. Mean effects of phosphorus and inoculation on grain yield, and interaction with year/location were estimated by fitting the following linear mixed model:

gy ∼ YL + P + I + P:I + YL:P + YL:I + trial

Where gy is grain yield, YL is the year/location factor, P and I are two-level factors for the application of phosphorus and inoculant, trial is a random factor for individual trials (farms) and “:” is an interaction term. We thereby ignored the higher-level, three-way, interaction between P, I and year/location. Significance was tested with a type-III ANOVA using the Satterthwaite approximation for the denominator degrees of freedom. For the four individual combinations of treatment levels, T, means, standard errors and Least Significant Differences (LSD) at the 5 percent level were calculated using the model:

gy ∼ YL + T + YL:T + trial

Mean separation of treatment groups within year/location was done at *P <* .05, using Tukey’s adjustment for multiple comparisons. For testing the effect of soil properties on response to P and I we fit the model:

gy ∼ P + I + P:I + X + X:(P + I) + (Z|YL/trial)

Where X is an individual soil property and (Z|YL/trial) is a random term accounting for year/location and trial, with Z being a random intercept for each level of YL and trial (Z = 1) or, alternatively, a set of YL and trial specific responses to P and I (Z = P + I). In the latter case, the interaction between X and P and I is corrected for location-specific responses to inputs.

## 3 Results and discussion

### 3.1 Soil properties

The soil textural classes were different among the Woredas, silt clay aAda’a and Gimbichu (Central Ethiopia), silty clay loam at Damote (Southern) and clay at Ginir (South East) and with low to moderate C contents (Table 3). Most of the farms had low average soil N content (Hazelton and Murphy, 2007) and very low average concentration of available P (Mallarino et al., 2013). The latter was moderate at Damote. The soil pH was neutral for all Woredas, except for Gimbichu where it was moderately alkaline. The CEC was moderate at all location, except Ada’a where CEC was very high (Hazelton and Murphy, 2007).

**Table 3:** Average soil properties of fields with demonstration trials on farmer’s plots at different Woredas.

Woreda	n	pH (H2O)	OC (%)	Total N (%)	P Meh. (mg kg^−1^)	Exchangeable cations (cmol_+_ kg^−1^)				Soil texture (%)			
						Ca	Mg	K	CEC	Sand	Silt	Clay	Class
Ada'a	19	6.6^a^	1.90^a^	0.13^a^	11.34^ab^	24.06^a^	3.97^a^	0.81^a^	45.62^a^	24	31	45	Silty clay
Damote*	27	6.7^a^	1.40^b^	0.13^a^	34.57^a^	16.87^a^	3.35^a^	3.29^b^	19.13^b^	32	29	39	Silty clay loam
Gimbichu*	7	7.7^b^	1.03^b^	0.12^a^	10.21^ab^	19.98^a^	2.08^a^	0.56^a^	22.69^b^	22	30	48	Silty clay
Ginir*	4	6.9^ab^	1.47^ab^	0.17^a^	4.79^b^	22.45^a^	4.08^a^	0.84^ab^	27.40^b^	38	22	40	Clay

Subscripts (within the columns) indicate differences in the soil properties across locations at the 0.05 level after Tukey adjustment for multiple.

* Values for % sand, silt and clay at 0–15 cm predicted (ISRIC 250 m soil property maps, www.soilgrids.org).

### 3.2 Chickpea grain yield response to P and/or I

Overall, the treatments significantly increased chickpea grain yield (p < .0001, Table 4 and Suppl. Fig. 1). Compared with the yield on the control plots, P and I increased chickpea grain yield by 413 and 335 kg ha^−1^, respectively. The combined effect of P + I averaged 604 kg ha^−1^, with no significant interaction between P and I (p = .1085, Table 4). The grain yield obtained with P + I was significantly different (P < .05) from the application of only one of the treatments. Grain yields with either P or I alone, though significantly different from the control and the P + I plots, were not statistically different from one another.

**Table 4:** Average chickpea grain yields (kg ha^−1^) for control (no inputs), P, I and P + I treatments in on-farm demonstration trials in different years/locations in Ethiopia. P = 23 kg P_2_O_5_ kg ha^−1^ applied as DAP, TSP or NPS; I = seeds inoculated with *Mesorhizobium* inoculum.

Year/Location	*n*	control	I	P	P + I	LSD (treatments within Year/Location)	SE
2012/Damote	17	1593^a^	2043^bc^	1951^b^	2194^c^	152	128
2013/Damote	3	1747	1796	2029	1843	ns	306
2014/Adaa	41	1937 ^a^	2272^b^	2197^b^	2548^c^	98	83
2014/Damote	25	2006^a^	2560^b^	2501^b^	3091^c^	125	106
2015/Adaa	4	1693^a^	2348^bc^	2089^b^	2453^c^	313	265
2015/Damote	7	1443^a^	1588 ^ab^	1746^bc^	1919^c^	237	200
2015/Gimbichu	6	1510^a^	2413^c^	1806^b^	2326^c^	256	216
2015/Ginir	4	958^a^	1170^ab^	1252^ab^	1349^b^	313	265
**Total**	107	1611^a^	2024^b^	1946^b^	2215^c^	88	74

Subscripts indicate the groups within location/year (the row) different at the 0.05 level after Tukey adjustment for multiple comparisons. ns = non-significant at P < .05. SE and LSD are the standard error of the means and the 0.05 LSD within year/location, respectively.

Year/location combinations differed significantly in terms of control yields (p < .0001) and response to both P and I (p < .0001), with a very small response observed in 2013/Damote-Gale and a large response in 2014/Damote-Gale (Suppl. Fig. 1). Average grain yield of farms with P + I treatment for individual year/locations were consistently greater than for the control plots. The lack of response in 2013 in Damote-Gale (Table 4) was an exception, which might be attributed to a small number of replicate fields. Comparing year/location results, the largest average grain yield (3091 kg ha^−1^) was obtained from 2014/Damote-Gale; the average grain yield in the control treatment (2006 kg ha^−1^) was also relatively large in this season and location. The relatively larger amount of total rainfall received and favourable distributions during the 2014 growing season presumably resulted in such large yields. On the other hand, the least yields of all the treatments were recorded in the 2015 growing season at Ginir. This location received in 2015 only 66% of the rainfall in normal years, with terminal drought occurring after flowering (Table 1). Moreover, uniformly poor chickpea grain yield was evident for all the experimental locations during 2015 as the country experienced the worst drought in history coinciding with a strong El Niño impact. Moisture availability is key for attaining high dry matter and seed yield in legumes (Singh and Rana, 2006; Walley, 1986). The influence of moisture stress on plant growth and legume-rhizobium symbiosis depends on the degree of the stress and the plant physiological growth stage (Zahran, 1999; Hungria and Vargas, 2000).

### 3.3 Nodulation, total N uptake and response to P and/or I

We were only able to estimate the size of the native rhizobium population in eight soils at Damote-Gale in 2012: in four of these we could not detect any rhizobia and in the remaining soils, the population was less than 10 cfu g^−1^ of soil. Although this was a small sample of soils, they were randomly selected from the 20 farmer’s plots studied in 2012 and are likely to be generally representative of the rhizobium population densities in this region.

Nodule counts were recorded on farms at Damote-Gale (2012 and 2014) and Adaa’ (2014) and overall inoculation enhanced the nodulation of chickpea, though the nodule counts vary in the different year/locations. Nodule counts of 22–48 per plant were recorded in the I and P + I treatments whereas this varied from 6 to 21 in non-inoculated control plots (data not presented). Overall, all treatments significantly improved the total N uptake in chickpea plants (Table 5), suggesting a well-functioning symbiosis. Inoculation, either alone or in combination with P, increased N uptake on average by 14–25 kg ha^−1^ as compared with the control (Table 5). The largest and significant N uptake was recorded from the P + I treatment while P and I were statistically on par. In contrast to the overall predicted mean, the N uptake across the different year/locations varied. However, for almost all years/locations, the smallest values were consistently recorded from the control plots. Increased N uptake recorded in plots with inoculation treatments were attributed to effective nitrogen fixation in inoculated plants. Chickpea is known to be specific for its rhizobial requirements (Tena et al., 2016; SPG, 2016) and generally responds well to inoculation (Ben Romdhane et al., 2008; Funga et al., 2016; Khaitov et al., 2016). MPN assessment in soil samples at representative farms in the study area resulted in low count of rhizobia compatible with chickpea. Inoculation is beneficial for the crop in soils where there is a small population of compatible rhizobia (Thies et al., 1991a, Thies et al., 1991b). Despite the observed variation in N uptake between individual year/locations, the relatively less uptake at specific locations (notably at Ginir/2015) is to be noted. Though data on nodulation status of chickpea plants growing at this specific site was not recorded, the terminal drought that occurred during the growing season might be a reason for the low N uptake (Table 1). It is generally known that drought seriously affects attachment of the bacteria to the root hairs, nodulation and N fixation in legumes (Zahran, 1999).

**Table 5:** Total N uptake by chickpea (kg ha^−1^) for control (no inputs), P, I and P + I treatments in on-farm trials in different years/locations in Ethiopia. P = 23 kg P_2_O_5_ kg ha^−1^ applied as DAP or TSP; I = seeds inoculated with *Mesorhizobium* inoculum.

Year/Location	n	control	I	P	P + I	LSD (treatments within Year/Location)	SE
2012/Damote	6	39.7^a^	77.9^c^	59.8^b^	94.8^d^	11.8	8.4
2013/Damote	1	42.5	46.2	61.5	62.6	ns	20.7
2015/Ada’a	4	69.3^a^	95.2^b^	81.0^ab^	91.8^b^	14.5	10.3
2015/Damote	5	67.0^ab^	60.4 ^a^	69.4 ^ab^	75.7^b^	12.9	9.2
2015/Gimbichu	7	60.7^a^	85.5^b^	65.6^a^	85.0^b^	10.9	7.8
2015/Ginir	5	42.0^a^	38.3^a^	40.6^a^	58.2^b^	12.9	9.2
Overall	28	53.5 ^a^	67.3 ^b^	63.0 ^b^	78.0 ^c^	6.7	4.8

Subscripts indicate the groups within location/year (row) different at the 0.05 level after Tukey adjustment for multiple comparisons. ns = no significant difference at P < .05. SE and LSD are the standard error of the means and the 0.05 LSD within year/location, respectively.

### 3.4 Soil properties and response to inoculation and phosphorus application

It is possible that different responses to P and I between different years/locations or individual fields are due to differences in soil properties. Nitrogen fixation by rhizobia is known to be affected by soil properties such as pH and N content, while the effectiveness of phosphorus fertilizer may be reduced by soil acidity (Hungria and Vargas, 2000; Andrade et al., 2002; Zerihun et al., 2015). We therefore tested the relationship between grain yield responses to inoculation and phosphorus application at individual farms and individual soil properties [pH, %N, %OC, CEC, K and Ca (cmol + kg^−1^)], with and without correction for year/location. Without year/location correction, inoculation response was significantly and positively affected by %N and %OC, while response to phosphorus fertilizer was positively affected by K. None of these relations were significant after correction for year/location, however, suggesting that the effect of soil properties on response to P and I is relatively weak and/or confounded with year/location.

**Fig. 2:**
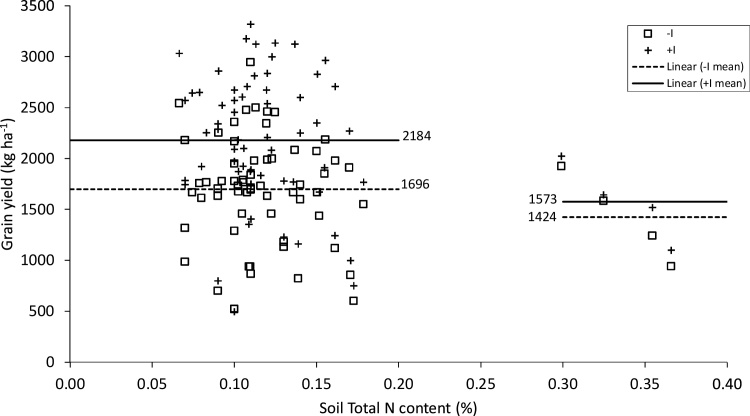
Relationship between total soil N content (%) and chickpea grain yield (kg ha^−1^) with and without inoculation.

Confounding is particularly apparent in the case of the effect of %N on inoculation response. Observed responses were considerably lower for a small set of fields with very high soil N contents (N > 0.25%) (left side of Fig. 2), compared to the majority of fields with N < 0.25%, the former having a mean response of only 149 kg ha^−1^ against 488 kg ha^−1^ for the latter (p < .05). In itself, this result is consistent with the expectation that the response to inoculation will be strongest in soils where N is limiting. However, the farms with large %N were all sampled in Damote in 2015, a year/location in which response to inoculation was relatively low on average (Table 4). This makes it impossible, given the small number of fields with N > 0.25%, to disentangle the effect of soil N from that of other unobserved year/location-level effects.

### 3.5 Variability in grain yield and responses to P and/or I

**Fig. 3:**
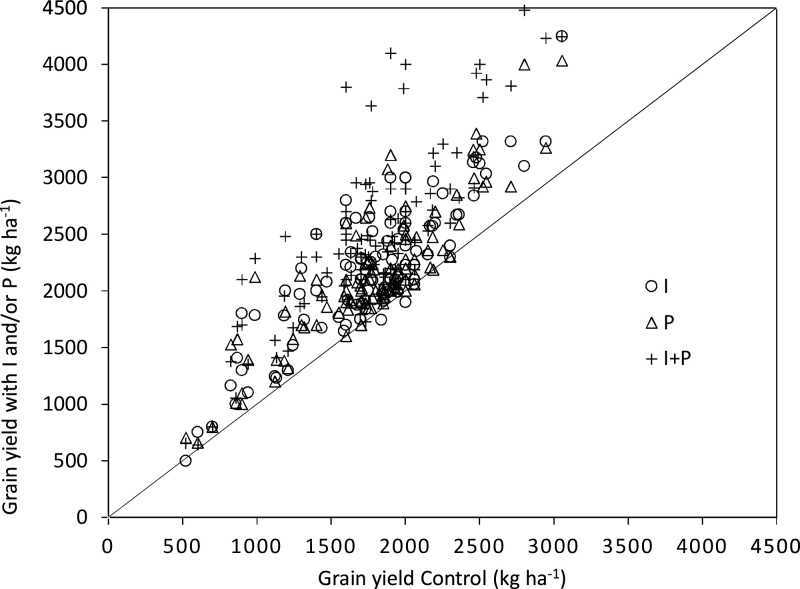
Chickpea grain yields control (kg ha^−1^) and response to P, I and P + I for individual farms in the target Woredas in Ethiopia (2012–2015). P = 23 kg P_2_O_5_ ha^−1^applied as TSP or DAP fertilizer; I = seed inoculated with *Mesorhizobium.*

Increased chickpea grain yield due to application of P and/or I was evident on most target farms, with only few exceptions where yields on inoculated plots were similar or inferior to those on the corresponding control plots (Fig. 3). However, grain yields on control plots and responses to the treatments on individual farms varied greatly. Thus, observed yield on control plots ranged from 521 kg ha^−1^ (2012/Damote-Gale) to 3054 kg ha^−1^ (2014/Damote-Gale), whereas the yields with P and/or I ranged from 640 kg ha^−1^ (2015/Ginir) to 4500 kg ha^−1^ (2014/Ada’a) (Fig. 3). These yields can be considered with reference to the national average yield, which is currently about 1800 kg ha^−1^ (CSA, 2016).

**Fig. 4:**
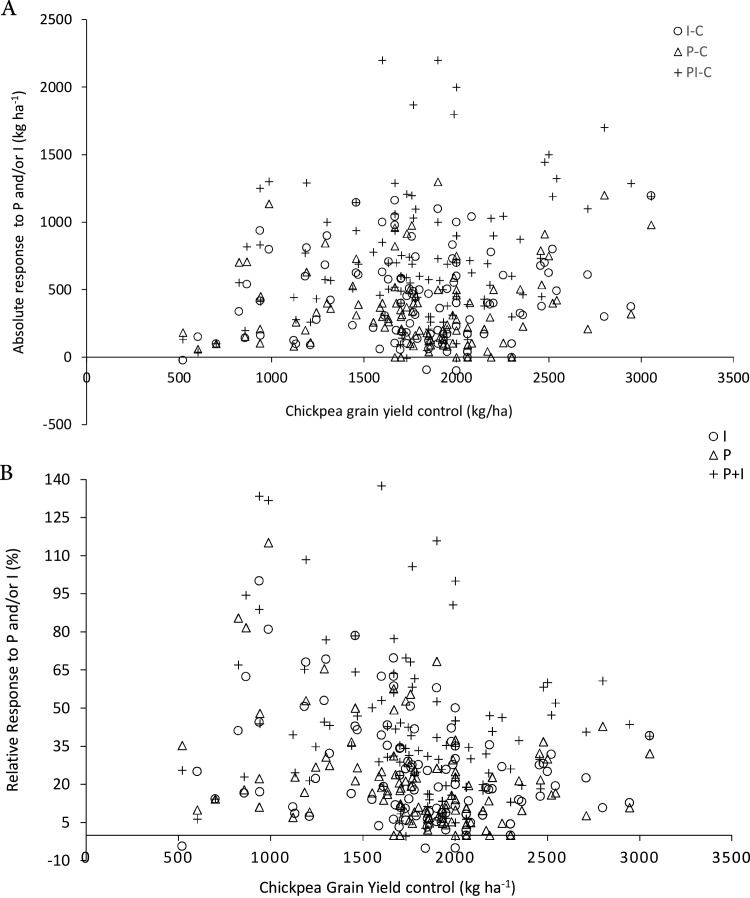
A Chickpea absolute grain yield (kg ha^−1^) in the control treatment and response to P, I and P + I for individual farms in the target Woredas in Ethiopia (2012–2015) as absolute yield (kg ha^−1^; yield of P and/or I minus control yield). B Relative response in grain yield of chickpea to P and/or I plotted against the control yield (%, yield of P and/or I minus control yield divided by control yield).

Absolute responses were relatively stable across yield levels. Farms with relatively small control yields (521–1800 kg ha^−1^), less than the national average, responded well to P and/or I, with absolute responses within the range of 400–2200 kg ha^−1^ (Fig. 4A). However, we did observe minimal yield responses on some of the farms which had very small control yields, with absolute responses less than 400 kg ha^−1^. Large absolute responses (1800–2200 kg ha^−^1) were recorded from the two major chickpea producing Woredas, Damote-Gale and Ada’a in 2014 on farms with control yields less than or equal to the national average. As a result of the relatively stable absolute responses, relative responses to P and/or I were larger for farms with small control yields, and decreased with increasing control yields (Fig. 4B). Relative yield responses up to 138% (i.e. more than double) the control yields, were recorded for a number of farmers with control yields less than the national average (<1800 kg ha^−1^). This indicates a clear opportunity to boost chickpea productivity on-farm using inoculant technology.

Given the variable responses to P and I observed in Damote over the four years (Suppl. Fig. 1), it seems that variation is not due to location specific constraints but rather due to random differences between years and individual farms.

### 3.6 Distribution of the responses to soil fertility treatments

**Fig. 5:**
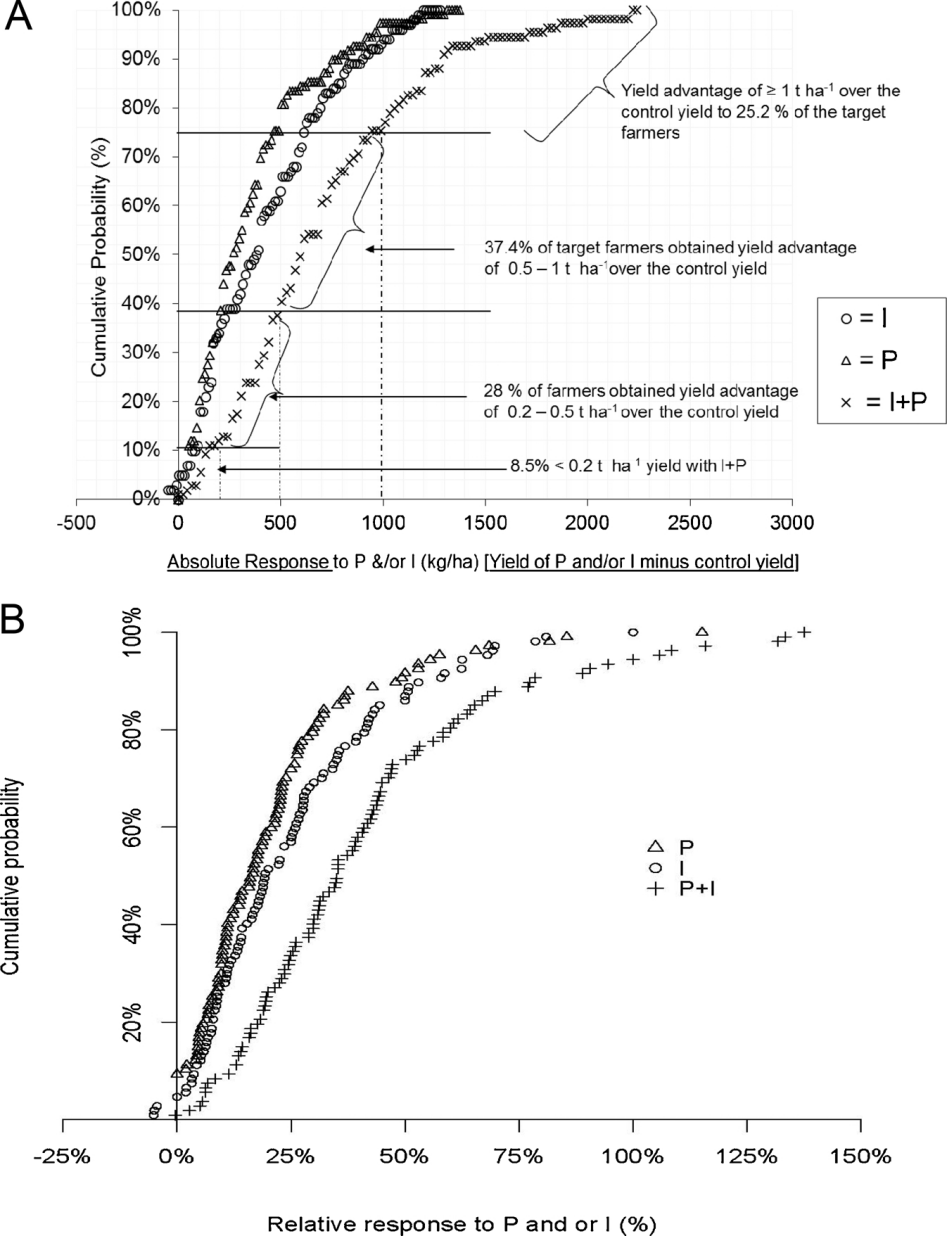
A Cumulative probability of absolute response (kg ha^−1^) in chickpea yield to P and/or I compared with the control treatment. B Cumulative probability of relative response (kg ha^−1^) in chickpea yield to P and/or I compared with the control treatme.

On-farm variability in yield response is a common phenomenon among smallholder farmers in sub-Saharan Africa (Vanlauwe et al., 2016; Ronner et al., 2016). Thus, it is important to look into the distribution of the responses and corresponding benefits due to a specific technology for its wider promotion. Our result indicated that application of the soil fertility treatments have resulted in increased chickpea yield (from at least 3% relative responses and up) for 99% of farms involved in this study, irrespective of differences in agro-ecological location (Fig. 5A and B). However, the frequency and magnitude of the yield response across farms greatly varied. Yield variations across farms is a risk or an opportunity for adoption of new technology by farmers. Good responses (over the control) should be visible on large number of farms at a given location so that farmers decide to take-up the new technology. Of the total number of target farms, 67% obtained 200 kg ha^−1^ or more extra grain yield (over the control yields) by the application of either P fertilizer or Inoculation alone while the same proportion of farmers produced 450 kg ha^−1^ or more with combined application of P and I (Fig. 5A). If the yield gain (over control yield) is increased to 1000 kg ha^−1^ or more, however, this could be achieved by 25% of the farmers applying both P + I, while only 0.8% of the farmers would achieved this if applying P or I (Fig. 5B). Assuming that a 10% yield increase is needed for treatment effects to be visible to farmers (Ronner et al., 2016), 71%, 73% and 92% of the farmers could achieve this (10% yield increase) with application of P, I, and P + I, respectively. However, in reality the larger the magnitude of the yield responses (the larger the amount of yield increased) and the higher the frequency (the more the number of farmers achieving this at a given location), the better the visibility of the benefit to farmers and the greater the chances for adoption of the technology.

### 3.7 Additive benefits and implication for adoption of inoculant technology

In the target farms, a yield response of 500 kg ha^−1^ or more over the control yield (absolute yield response) attained with Inoculation and P fertilizer application by 63% of the farmers (Fig. 5A). This response represents 35% or more yield increase (relative response) (Fig. 5B). By contrast, the same level of yield could be achieved by 25% of farmers by inoculation and by 14% of farmers with P application. Assuming a cost benefit of 4000 birr (200 USD) per ha calculated in earlier studies (van den Broek et al., 2014) in local chickpea market in Ethiopia for a yield of 1480 kg ha^−1^, an absolute response of 500 kg ha^−1^ would represent 1350 birr (60 USD) additional household income per ha. However, triple price increase for chickpea since 2014 is to be noted, thus resulting in increased benefit to smallholders currently. The benefit would be even more for 25% of the farmers who obtained >1000 kg ha^−1^ yield over the control because of combined application of P and I (Fig. 5A). While further studies of the adoption of inoculant technology and marketing of chickpea grain are needed, and currently underway with support from the N2Africa project and others, the increasing popularity of inoculants use in legume production in Ethiopia is evident. During the last four years (2012–2015), since the launching of N2Africa project in Ethiopia, inoculant production by a private company – Menagesha Bio-tech Industry (MBI) has expanded six-fold (from 28000 to 165000 sachets annually), while the distribution and sales of inoculants has risen by seven and 13-fold, respectively (Ampadu-Boakye et al., 2017), where chickpea inoculants comprise a substantial proportion of sales. The additive value of inoculation for chickpea production and its cost effectiveness has been well recognized by smallholders and its wider adoption is to be expected.

## 4 Conclusion

On a wide range of on-farm trials, covering diverse agro-ecological locations over four regions in Ethiopia, inoculation and P fertilizer application increased chickpea grain yields. Despite considerable and seemingly random variation, the response to these inputs was consistently positive. Yields were greatest when both I and P were applied together in combination, with an average increase of 38% over the control plots. No compatible chickpea rhizobia were detected in half of the soil samples tested while the population was less than 10 cfu g^−1^ of soil in the remainder, explaining why inoculation benefits chickpea growth and yield. Thus, inoculation, alone or in combination with P, enhanced the nodulation of chickpea, stimulated nitrogen fixation and increased N uptake. This is the first time that the benefits of rhizobial inoculation for enhanced nitrogen fixation and yield of chickpea have been demonstrated across large numbers of smallholder farms in Africa. Together with small amounts of P fertilizer, rhizobial inoculation offers a cheap and highly effective means for the sustainable intensification of smallholder agriculture.
