# Supplementary material for: Additive yield response of chickpea (Cicer arietinum L.) to rhizobium inoculation and phosphorus fertilizer across smallholder farms in Ethiopia
Source: Agric Ecosyst Environ. 2018 Jul 1;261:144–52. doi: 10.1016/j.agee.2018.01.035 (PMC5946702; doi:10.1016/j.agee.2018.01.035)
Supplement: Supplementary file 1 [file mmc1.docx]

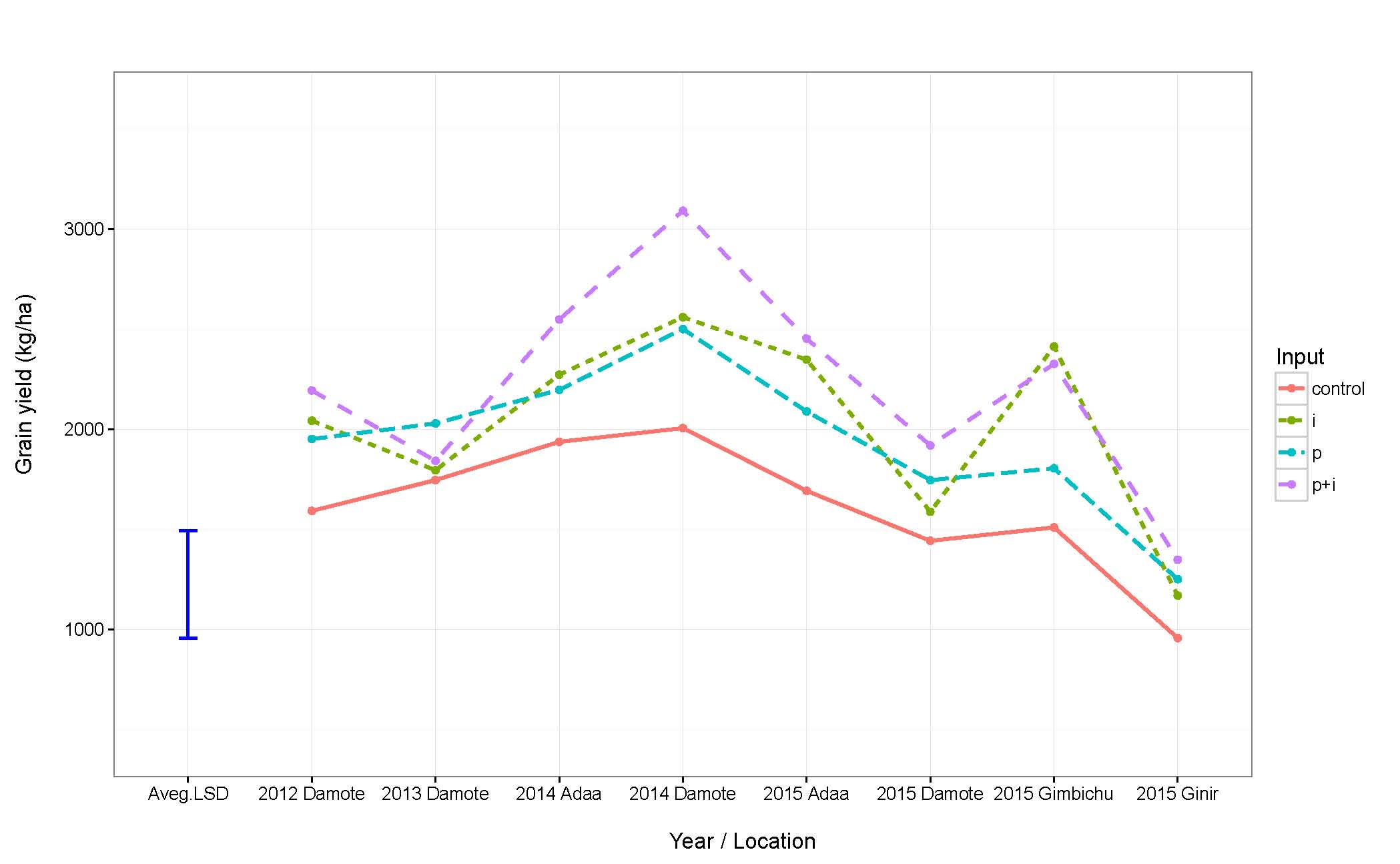


Supplementary Fig. 1: Predicted means for chickpea grain yield (kg ha^-1^) with P, I and P+I applications at different year/Woredas in Ethiopia (average LSD bar 5%)
